# Beyond Burnout: Rebuilding Medical Professionalism in Europe's Overstretched Health Systems

**DOI:** 10.7759/cureus.112259

**Published:** 2026-07-08

**Authors:** Gian Marco Rizzuti

**Affiliations:** 1 Orthopaedics and Traumatology, Bozen Central Hospital, Bozen, ITA

**Keywords:** burnout, health policy, medical education, medical professionalism, physician workforce, professional identity, quality improvement, surgical training, workforce crisis

## Abstract

Medicine in Europe is undergoing a profound cultural and structural transition. Public debate often describes the current crisis in terms of burnout, workforce shortages, generational change, or financial pressure. Although these factors are important, they do not fully explain the erosion of professional satisfaction, clinical confidence, and institutional trust among physicians.

This editorial argues that the current debate should move beyond burnout as an individual occupational syndrome and toward a broader understanding of medical professionalism as a fragile institutional compact. What distinguishes this perspective from previous discussions is its focus on the relationship between responsibility and the structures that make responsibility sustainable: training, mentorship, autonomy, protected supervision, psychological safety, and institutional loyalty.

The problem is especially visible in surgical and hospital-based disciplines, where professional identity is built through progressive responsibility, meaningful clinical exposure, and shared accountability. When physicians are asked to endure pressure without adequate formation or support, the result is not resilience but disengagement, defensive practice, and loss of meaning.

European medicine does not need a return to the old mythology of the physician as martyr, but neither can it survive as a purely transactional form of shift work. A renewed professional compact is needed: one that preserves high standards, ethical commitment, and clinical presence while recognising human limits and institutional responsibility. Rebuilding this compact is essential if healthcare systems are to retain not only their workforce but also the professional ethos that makes safe and humane care possible.

## Editorial

Medicine is again being asked to define what kind of profession it wants to be. Across Europe, hospitals are under financial pressure, health systems face shortages of physicians and nurses, and many doctors describe an increasing loss of meaning in daily clinical work. The vocabulary of the crisis is now familiar: burnout, workforce shortage, administrative overload, loss of autonomy, and unsustainable working conditions.

These words are accurate, but they may still be insufficient. Burnout describes what happens to many physicians, but not always why the profession itself feels increasingly unstable. Workforce shortage describes the lack of personnel, but not why trained professionals leave or psychologically withdraw from clinical work. Financial pressure explains many institutional decisions, but not the cultural exhaustion that now affects the relationship between physicians, hospitals, patients, and society.

In two previous editorials, I discussed separate aspects of this problem: first, the misleading opposition between medical vocation and work-life balance, particularly in surgical training, and second, the wider European implications of the German hospital crisis [1,2]. The present editorial brings these threads together and advances a broader argument: the current crisis is not only a crisis of burnout or funding but a crisis of the professional compact that once made medical responsibility sustainable.

For decades, medicine was framed as a vocation. Long hours, personal sacrifice, and constant availability were treated as signs of moral seriousness. This culture produced dedication and resilience, but it also normalised forms of endurance that are difficult to defend today. Younger physicians are now asking for protected time, psychological sustainability, predictable working conditions, and a life outside the hospital. In some professional debates, this is interpreted as a decline in vocation.

That interpretation is too simple. Many younger physicians are not rejecting responsibility; they are rejecting responsibility without preparation, supervision, recognition, or institutional loyalty. There is a difference between sacrifice that has clinical and educational meaning and endurance that merely compensates for organisational failure. A 24-hour shift spent learning, deciding, operating under supervision, and gradually acquiring autonomy is not the same as a 24-hour shift spent absorbing systemic inefficiency. The first may form a physician. The second may only exhaust one.

The literature on burnout supports this distinction. Physician burnout has been associated with emotional exhaustion, depersonalisation, reduced professional accomplishment, adverse effects on patient care, and consequences for workforce retention [3]. Shanafelt and colleagues have argued that addressing burnout requires action at the level of the healthcare system, not only at the level of individual resilience [4]. This is crucial. If burnout is treated only as a personal psychological problem, the proposed solutions will remain limited to coping strategies. If it is recognised as a systemic signal, the focus must shift toward work design, staffing, leadership, autonomy, and professional culture.

Burnout is therefore not merely the disease; it is also a symptom. It indicates that the internal architecture of medical professionalism is under stress. Medicine requires more than technical competence. It requires attention, judgement, empathy, responsibility, and the ability to remain present in situations of uncertainty and suffering. When systems repeatedly erode these capacities, they do not simply harm physicians as workers. They weaken the moral and cognitive conditions of safe care.

This problem is particularly visible in surgical and hospital-based disciplines. Surgical identity is formed through exposure, repetition, mentorship, and progressive autonomy. The operating room, emergency department, ward, and night shift are not only places of service provision; they are formative spaces. When these spaces are reduced to labour coverage without structured learning, training becomes hollow. Conversely, when young physicians are protected from responsibility for too long and then suddenly expected to function independently, anxiety replaces confidence.

Recent survey data from young Italian surgeons illustrate this mismatch. Molteni et al. reported that many residents and early-career surgeons perceived insufficient operative exposure and inadequate preparation for real-world surgical demands [5]. The central problem was not simply that young doctors were unwilling to work. It was that training pathways did not reliably transform time in the hospital into competence. Presence without formation is not professionalism; it is attendance.

Although these data come from the Italian surgical context, their relevance is broader. Similar tensions between service provision, supervised autonomy, workload, and perceived preparedness are increasingly recognisable across European hospital systems. The Italian example should therefore be read not as an isolated national anomaly, but as a useful lens through which to examine a wider challenge in postgraduate medical training.

Another neglected dimension is the psychological burden of adverse events. Wu introduced the concept of the "second victim" to describe clinicians harmed by the emotional consequences of medical error [6]. Scott et al. later described recovery trajectories among healthcare professionals involved in adverse patient events, highlighting the need for support rather than silence [7]. This concept remains essential for understanding professional sustainability. A system that asks physicians to make difficult decisions must also provide structures to process complications, errors, and moral distress. Otherwise, accountability becomes isolation.

Psychological safety does not mean lowering standards. On the contrary, it makes honest accountability possible. Morbidity and mortality meetings, peer support, mentorship, structured debriefing, and non-punitive safety cultures are not signs of weakness. They are professional infrastructure. Without them, physicians may respond to adverse events by withdrawing from complexity, practising defensively, or leaving high-risk specialties altogether.

The European context makes this discussion urgent. The Organisation for Economic Co-operation and Development (OECD) and European Commission reported that 20 EU countries experienced doctor shortages in 2022 or 2023, while 15 reported nurse shortages; using minimum staffing thresholds, the EU shortage of doctors, nurses, and midwives was estimated at approximately 1.2 million in 2022 [8]. WHO Europe has similarly warned that health and care workforce challenges require strategic policy responses across the region [9]. These numbers matter, but they do not tell the whole story. Producing more physicians will not solve the crisis if health systems remain unable to train, support, and retain them.

Recruitment without retention is a delay mechanism. International recruitment may fill immediate gaps, but it can also shift shortages from one country to another. Digitalisation may reduce some inefficiencies, but it cannot replace mentorship, clinical judgement, or institutional trust. Hospital reform may be necessary, but reform that is perceived only as rationing or acceleration will deepen cynicism rather than restore confidence.

What is needed is a renewed professional compact. Such a compact should begin with a simple principle: responsibility must be matched by preparation. Physicians should not be asked to carry high-risk clinical responsibility without adequate supervision, structured training, and progressive autonomy. Training should be evaluated not only by years completed or hours worked but by competence acquired.

Second, sustainability should be recognised as a professional requirement, not as a generational luxury. Safe care depends on physicians who are alert, supported, and capable of judgement. Human limits are not the enemy of professionalism; they are one of its conditions.

Third, autonomy must be protected. Clinical judgement cannot be reduced entirely to administrative compliance, coding systems, productivity metrics, or economic pressure. Medicine requires accountability, but accountability is not the same as managerial control. Physicians must remain moral agents, not only service providers.

Fourth, adverse events must be handled through learning cultures rather than fear cultures. The second victim phenomenon should be recognised within hospitals as a patient safety and workforce issue. Supporting clinicians after adverse events is not sentimental; it is part of maintaining a competent and honest profession.

Finally, healthcare reform must be grounded in trust. Trust is not an abstract value. It is the invisible infrastructure that allows patients to accept care, young physicians to learn, senior physicians to teach, and institutions to change without being perceived as hostile. When trust collapses, every reform becomes another threat.

In practical terms, these principles could be translated into several institutional and policy-level initiatives. Hospitals and training bodies could introduce protected operative and clinical exposure targets that measure competence rather than mere attendance, structured mentorship programmes for residents and early-career physicians, mandatory debriefing pathways after adverse events, protected time for supervision and teaching within senior physicians' workload, and institutional indicators that evaluate training quality, psychological safety, and physician retention alongside productivity and financial performance. At the policy level, workforce planning should therefore move beyond recruitment numbers and include measurable standards for training environments, supervision capacity, and professional sustainability.

The future of European medicine should reject two extremes. It should reject the old mythology of the physician as martyr, endlessly available and silently suffering. It should also reject the reduction of medicine to a purely transactional occupation emptied of ethical depth. The physician is neither a saint nor a technician. The physician is a trained professional whose work requires competence, responsibility, judgement, and humanity. This view is consistent with organisational literature describing professionalism as a distinct logic of work that cannot be reduced entirely to market mechanisms or bureaucratic control [10].

The question is no longer whether medicine is a calling or a job. It must be both: a profession with ethical depth and human limits. Future research should examine which institutional interventions most effectively preserve professional identity, reduce avoidable burnout, and improve retention among early-career physicians. Future policy development should similarly focus not only on workforce numbers but also on the quality of training environments, supervision, autonomy, and support after adverse events. If European health systems fail to rebuild the structures that make this possible, the loss will not be only generational or economic. It will be professional.
